# In Vitro Analysis of Enamel Abrasion Using a Novel Manual Nano-Bristle Toothbrush: An Atomic Force Microscopy Study

**DOI:** 10.7759/cureus.75243

**Published:** 2024-12-06

**Authors:** Mohan Prasad, Prem Blaisie Rajula, Ravishankar PL, Sunanda Rao, Gayathri K, Mounika V, Murali Venkata Rama Mohan Kodali, Kalaivani V, Gracelin Soloman, Nelofar Nisha

**Affiliations:** 1 Periodontology, SRM Kattankulathur Dental College and Hospital, SRM Institute of Science and Technology, Chengalpattu, IND; 2 Oral and Maxillofacial Surgery, King Faisal University, Al-Ahsa, SAU

**Keywords:** atomic force microscopy, brushing simulator, enamel abrasion, nanobristle toothbrush, toothbrush bristle stiffness

## Abstract

Introduction: To evaluate the enamel abrasion effects of soft, ultra-soft, and nano-bristle toothbrushes using atomic force microscopy (AFM) to guide toothbrush selection for optimal enamel preservation.

Methods: This in vitro study involved 45 extracted human teeth (central and lateral incisors), randomly assigned to three groups (n=15 each): Group I (nano-bristle), Group II (ultra-soft bristle), and Group III (soft bristle). Each specimen underwent 10,000 brushing cycles with a standardized 2 N force to simulate one year of brushing. A slurry of commercially available toothpaste mixed with saline was applied, and brushing was performed with a mechanical brushing simulator. AFM analysis measured enamel surface roughness before and after brushing.

Results: Nano-bristle toothbrushes caused a minimal increase in surface roughness (mean change: 4 nm; p = 0.001), significantly less than the increases seen with soft (mean change: 14.08 nm; p = 0.001) and ultra-soft (mean change: 14.86 nm; p = 0.001) bristle toothbrushes. AFM analysis confirmed that both soft and ultra-soft bristles caused greater enamel abrasion compared to nano-bristles, with no significant difference between soft and ultra-soft bristle groups.

Conclusion: Nano-bristle toothbrushes demonstrated the least enamel abrasion in this study, suggesting they may provide a gentle alternative for preserving enamel integrity.

## Introduction

Dental plaque, a sticky film that forms on teeth, is a primary culprit in a range of oral health problems, such as dental caries, gingivitis, and periodontitis. As a result, effective plaque control is central to preventive oral healthcare [1,2]. Mechanical plaque control is widely regarded as the most common and effective method for patients to remove plaque at home, with daily brushing and the use of other oral hygiene tools being the most reliable way to achieve the benefits of plaque removal. However, toothbrush use has also been linked to potential damage to both soft and hard oral tissues, as well as tooth sensitivity [3]. For this reason, dental professionals increasingly recommend toothbrushes with soft bristles.

Good oral hygiene is essential for general health, and choosing the correct toothbrush is critical for both efficient tooth cleaning and enamel protection. According to bristle diameter, toothbrushes are often divided into three categories: soft (0.2 mm), medium (0.3 mm), and hard (0.4 mm) [4, 5]. Manual toothbrushes featuring soft conical filaments are claimed to provide better cleaning of subgingival pockets compared to a standard reference toothbrush approved by the American Dental Association (ADA) [ 6]. However, studies have shown conflicting results regarding the impact of bristle stiffness on enamel abrasion. Some research suggests that hard toothbrushes cause greater abrasion compared to soft brushes [7,8]. Conversely, other research indicates that soft brushes may result in greater abrasion than hard brushes [9,10]. This discrepancy can be attributed to the flexibility of soft bristles, enabling them to cover a larger surface area and retain a greater amount of toothpaste.

Various in vitro investigations have utilized diverse methodologies to evaluate enamel surface abrasion. There has been a growing focus on the application of atomic force microscopy (AFM) in biological studies. AFM provides a high-resolution method for analyzing the surface morphology of biological specimens in standard environmental circumstances. The AFM microscope functions by detecting the surface using a sharp silicon tip on a micromachined probe, systematically scanning the surface in a linear fashion. Multiple operational modes are available; however, tapping mode AFM (TM-AFM) has demonstrated efficacy in examining enamel crystals, enamel dissolution, and the development of the acquired pellicle layer on enamel [11-13].

Recently, nano-bristle toothbrushes have been introduced. The manufacturers of the nano-bristle brush used in this study (Vi Brush Nano Bristles Toothbrush, Vi Brush India) state that their product has an innovative design that promises gentle and more effective cleaning, with potentially less risk of enamel damage. This toothbrush is said to contain around 12,000 ultra-fine bristles, approximately 80 microns in diameter. The manufacturer also states that the dense bristle arrangement creates a cushion-like texture, enhancing plaque removal even in difficult areas. Additionally, they claim that the soft, delicate bristles are designed to minimize abrasion, providing a protective and gentle approach for both enamel and soft tissues [14].

To date, no studies have evaluated the effectiveness of commercially available nano-bristle toothbrushes on enamel abrasion. This study hypothesizes that nano-bristle brushes cause less enamel abrasion than soft and ultra-soft brushes. Therefore, this study sought to evaluate the enamel abrasion caused by soft, ultra-soft, and nano-bristle toothbrushes using AFM. By evaluating the degree of abrasion these different bristle types cause, we can provide better guidance on selecting the most suitable toothbrush for maintaining enamel integrity while ensuring optimal oral hygiene.

## Materials and methods

The present in vitro study was conducted in the Department of Periodontics, SRM Kattankulathur Dental College, Potheri, Tamil Nadu, India.

Specimen collection

The institutional ethics committee approved the protocol (SRMIEC- ST0724-1499) on 31August 2024 for this in vitro study. Following the acquisition of written informed consent and a verbal explanation regarding the use of the research, periodontally compromised upper and lower incisor teeth were extracted and collected from patients and subsequently immersed in 10% formalin solution for a duration of two weeks. A sample size of 45 teeth was calculated using G*Power software (version 3.1, University of Düsseldorf, Düsseldorf, Germany), with an effect size of 1.0, a power of 95%, and a significance level of 5% (p < 0.05) [15,16].

Specimen preparation

**Figure 1:**
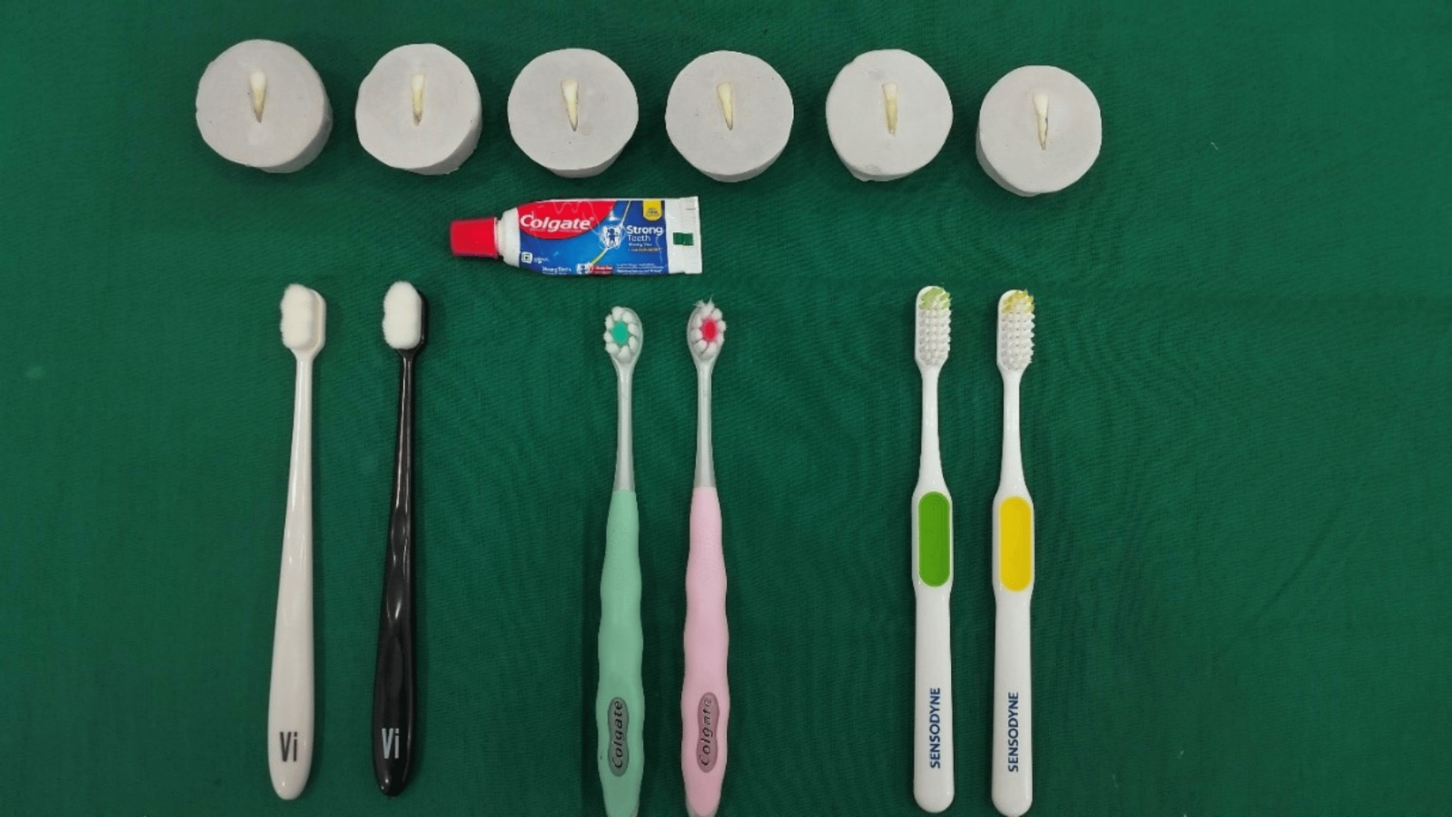
Toothbrushes used in the study From left: Group I, nano-bristle (Vi Brush Nano Bristles Toothbrush, Vi Brush India); Group II, ultra-soft bristle (Colgate Gentle Ultrafoam Toothbrush, Colgate-Palmolive (India) Ltd., ); Group III, soft bristle (Sensodyne Daily Care Soft Toothbrush, Schiffer & Menezes India Pvt. Ltd.)

The collected teeth were thoroughly examined for any surface defects, and those with conditions such as hypoplasia, fluorosis, cracks, caries, fractures, decalcification, or restorations were excluded. Before use, the teeth were subjected to ultrasonic cleaning, dried, and affixed to die stone to ensure proper fitting in the sample holders used during the brushing simulation (Figure 1). The teeth were subsequently allocated at random into three groups of 15 specimens each, depending on the type of toothbrush bristles used: Group I, nano-bristle (Vi Brush Nano Bristles Toothbrush, Vi Brush India); Group II, Ultra-soft bristle (Colgate Gentle Ultrafoam Toothbrush, Colgate-Palmolive (India) Ltd); Group III, soft bristle (Sensodyne Daily Care Soft Toothbrush, Schiffer & Menezes India Pvt. Ltd). To maintain consistency, all toothbrushes (nano-bristle, ultra-soft, and soft variants) were standardized in terms of handle and bristle material (nylon). This ensured that variations in enamel abrasion could be attributed exclusively to differences in bristle stiffness. Baseline surface roughness values were recorded using an atomic force microscope (AFM).

Brushing experiment

**Figure 2:**
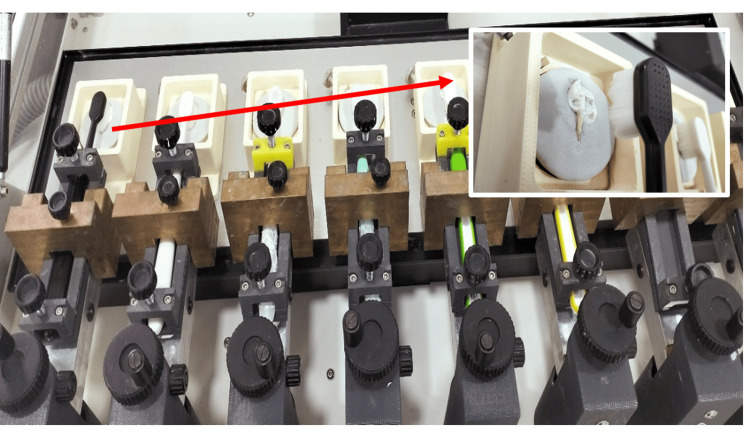
Set-up of SD Mechatronik (SD Mechatronik GmbH, Feldkirchen-Westerham, Germany) for brushing simulation with a close-up view of the nano-brush and attachment in the brushing simulator

Each specimen underwent 10,000 brushing cycles to simulate approximately one year of brushing. The buccal or labial surfaces(more prone to damage due to vigorous toothbrushing) of the crowns were brushed using the SD Mechatronik Zm3.8 automatic brushing simulator (SD Mechatronik, GmbH, Feldkirchen-Westerham, Germany). The brushing simulation involved a combination of linear (5,000 cycles along the Y-axis) and circular (2,500 clockwise cycles and 2,500 counterclockwise cycles) movements to replicate typical brushing patterns (Figure 2) [16]. The toothbrush heads were aligned parallel to the tooth surface, with a 200-gram weight applied at the center of the holder to exert a brushing force of 2 N.

A commercially available toothpaste with an RDA value of 68, formulated with fluoride, detergents, and abrasives, was mixed with normal saline at a 1:3 ratio to create a slurry, following the EN ISO 11609:2010 standard for toothpaste testing. The slurry was delivered at a flow rate of 10 ml/min, with fresh slurry applied after every 1,000 brushing cycles. Following each brushing session, the teeth were taken out of the holders, rinsed three times with distilled water, and gently wiped with cotton in the brushing direction without applying pressure. Furthermore, distilled water was used for brief ultrasonic cleaning procedures (one minute), followed by a final rinse with distilled water and air drying.

Sample preparation for AFM

**Figure 3:**
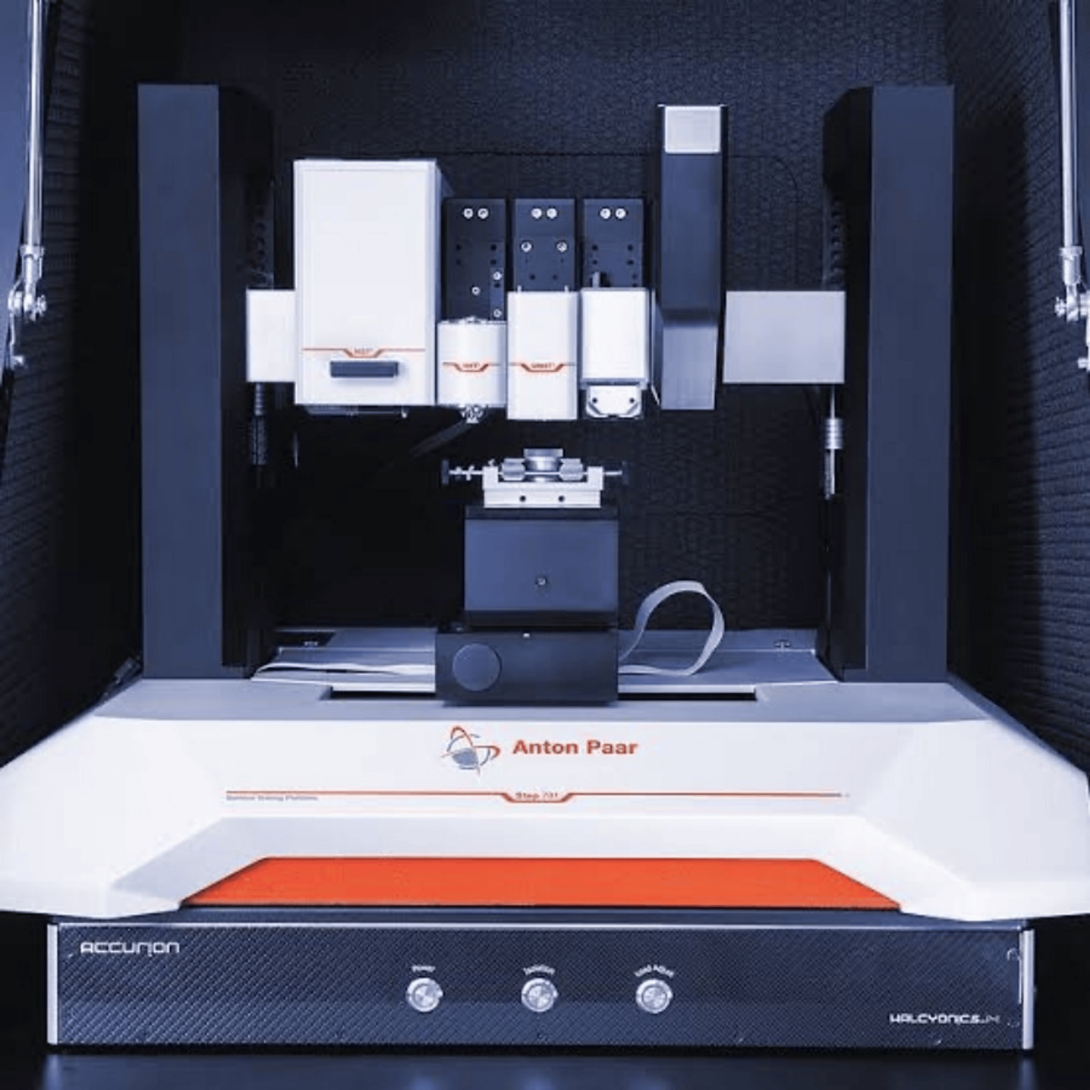
Atomic force microscope Anton Paar Step 700 (Nanosurf, Liestal, Switzerland)

The samples for AFM analysis were prepared following brushing treatment. The preparation involved thorough washing with double distilled water followed by natural drying on paper. The components were subsequently affixed to a designated support, ensuring that the examined surface faced upward. The pre- and post-roughness measurements were conducted utilizing the Anton Paar Step 700 (Nanosurf, Liestal, Switzerland) equipped with a Stat0.2LAuD cantilever, featuring a tip diameter of 1 nm, in static mode (Figure 3). The AFM was calibrated using a certified standard to adjust Z-axis (height) and X-Y (lateral dimensions) scaling by scanning known features and aligning the measurements with their true values, followed by routine validation to ensure accuracy. The pointed tip provides stability to prevent unintended movement of the surface. Image sizes encompass areas measuring 10μm × 10μm, with a lateral resolution of approximately 100 nm.

Statistical analysis

Data analysis was conducted using Jamovi version 2.6 (The Jamovi Project, Sydney, Australia). Descriptive statistics, including mean values and standard deviations, were calculated for surface roughness before and after brushing for each toothbrush type. A Shapiro-Wilk test was performed to assess normality, followed by a paired t-test to compare pre- and post-brushing surface roughness within each group. To evaluate differences in post-brushing surface roughness among the three toothbrush types, a one-way analysis of variance (ANOVA) was performed, with Tukey’s post-hoc test applied for pairwise comparisons. ANOVA assessed overall variance between groups, while the post-hoc test identified specific differences between toothbrush types. A p-value of 0.05 or less was considered statistically significant.

## Results

Table 1 displays the mean surface roughness (measured in nanometers) of three types of toothbrushes (nano bristle, soft bristle, and ultra-soft bristle) before and after brushing. The nano-bristle toothbrush showed a slight increase in surface roughness, with a pre-brushing mean of 13.5 ± 0.93 nm and a post-brushing mean of 17.4 ± 1.31 nm. The mean difference of 4 nm was statistically significant, as indicated by a p-value of 0.001, demonstrating that brushing with the nano bristle brush resulted in a modest increase in surface roughness. In contrast, both the soft-bristle and ultra-soft bristle toothbrushes exhibited a more pronounced increase in surface roughness. The soft bristle toothbrush had a pre-brushing mean of 13.5 ± 1.17 nm, which increased significantly to 28.3 ± 0.94 nm after brushing, with a mean difference of 14.08 nm and a p-value of 0.001, indicating a significant change. Similarly, the ultra-soft bristle toothbrush resulted in an increase from 13.3 ± 1.07 nm to 27.3 ± 1.10 nm, with a mean difference of 14.86 nm and a p-value of 0.001.

**Table 1:** Comparison of pre and post-brushing surface roughness using different toothbrush types Mean ± SD; Paired t test; *(p<0.05)

Bristle type	Surface roughness (nm)			95% confidence interval		
	Pre-brushing	Post-brushing	Mean difference	Lower	Upper	p-value
Nano bristle	13.5 ± 0.93	17.4 ± 1.31	-4	-5.06	-2.93	0.001*
Ultra soft bristle	13.3 ± 1.07	27.3 ± 1.10	-14.86	-15.4	-14.32	0.001*
Soft bristle	13.5 ± 1.17	28.3 ± 0.94	-14.08	-14.91	-13.24	0.001*

**Figure 4:**
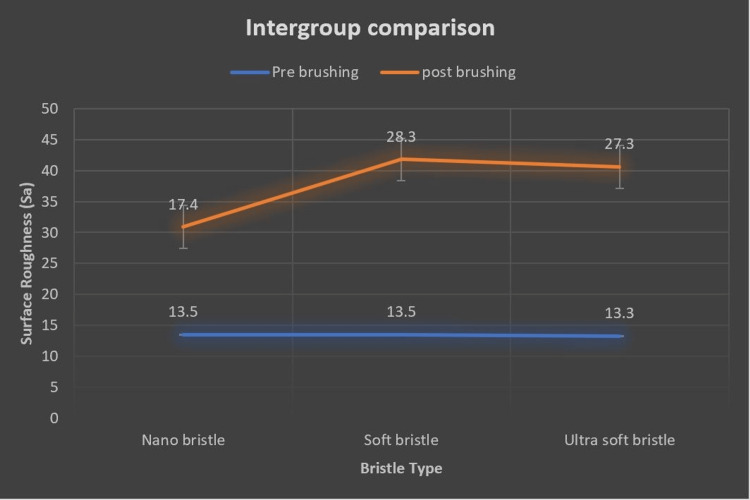
Inter-group comparison of surface roughness using atomic force microscopy

Both Table 2 and Figure 4 illustrate the mean differences in post-brushing surface roughness (in nanometers) between different types of toothbrushes (nano bristle, soft bristle, and ultra-soft bristle) using a one-way ANOVA with Tukey post-hoc test. The findings indicated a considerable disparity in surface roughness between nano bristle and soft bristle brushes, with a mean difference of 10.9 nm. This negative value indicates that the soft bristle toothbrush caused a significantly higher increase in surface roughness compared to the nano bristle. The p-value is highly significant (p < .001). Similarly, the difference between nano-bristle and ultra-soft bristle toothbrushes was also significant, with a mean difference of 9.90 nm. This shows that the ultra-soft bristle brush caused a significantly higher increase in surface roughness compared to the nano bristle, again with a highly significant p-value (p < .001). However, the mean difference in surface roughness between the soft bristle and ultra-soft bristle brushes was 0.98 nm, which was not statistically significant, indicating that both soft and ultra-soft bristle brushes had a similar impact on increasing surface roughness.

**Table 2:** Analysis of mean differences in post-brushing surface roughness between toothbrush types One-way ANOVA with Tukey post hoc test *** p < .001.

Bristle type	Nano bristles	Soft	Ultra soft
Nano bristle	—	-10.9***	-9.90***
Ultra-soft bristle	—	—	—
Soft bristle	—	—	-0.98

**Figure 5:**
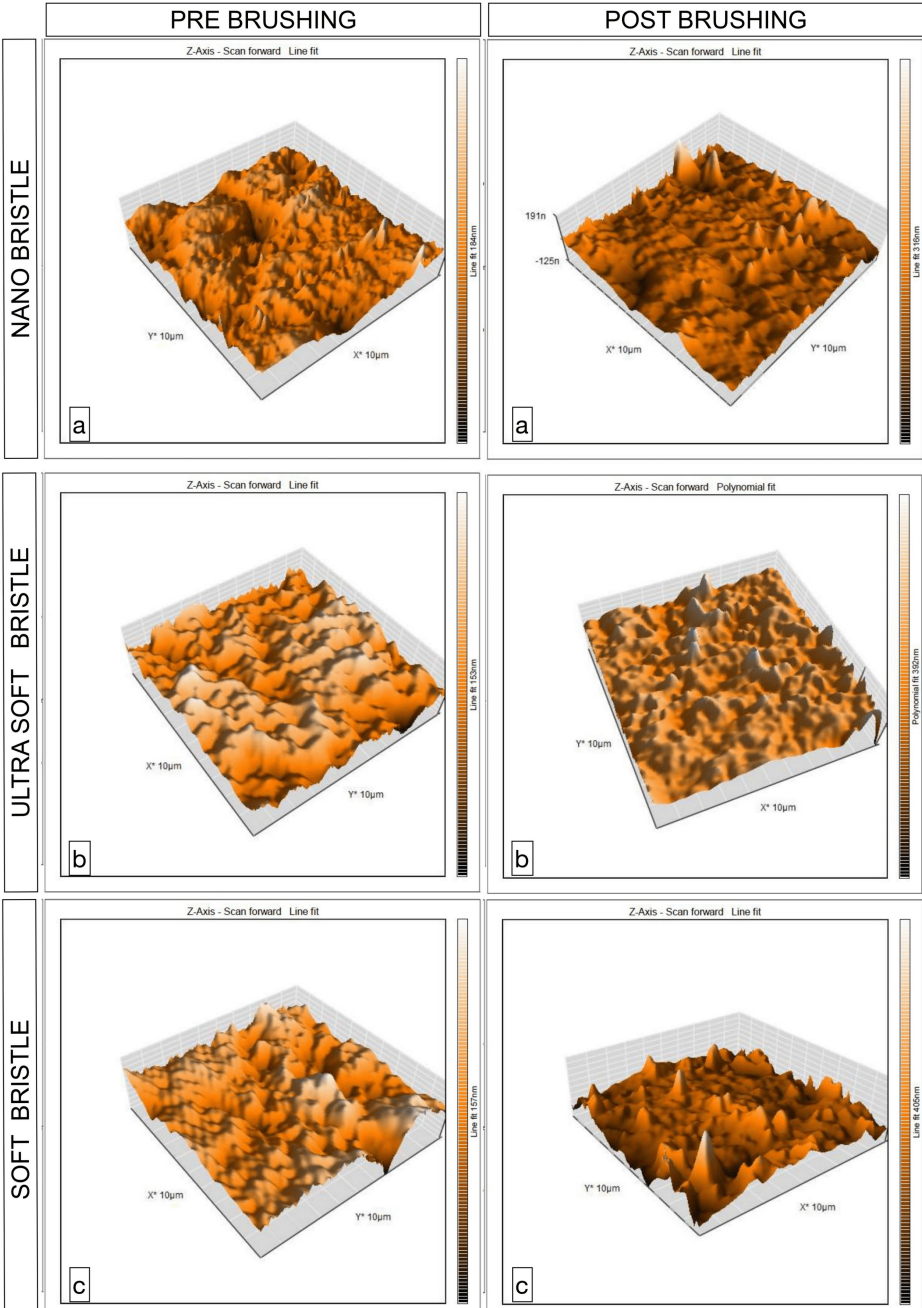
Atomic force microscopy images of the surface roughness produced in the three groups before and after brushing (a) Nano bristle;  (b) ultra-soft bristle; (c) soft bristle

The AFM images showed the greatest crest and trough in the soft and ultra-soft bristle group compared to the nano bristle group (Figure 5). In comparison to soft and ultrasoft toothbrushes, the nano-bristle toothbrush had the lowest mean roughness value (Sa) measured by AFM.

## Discussion

Oral home care is a key priority for dental professionals, as most periodontal diseases stem from bacterial infections that form biofilms. Since its invention, the toothbrush has been the most widely used tool for maintaining oral hygiene [1]. While manual toothbrushes are the cornerstone of daily oral care, their use is not without drawbacks. They have been associated with soft and hard tissue damage, as well as tooth sensitivity, particularly when used improperly or with excessive force [3]. Various factors have been identified as contributors to toothbrush abrasion, including brushing technique, force, duration, frequency, and, notably, the stiffness of the brush bristles [7].

Bristle stiffness plays a critical role in toothbrush performance. It is well-accepted that hard-bristled toothbrushes induce greater abrasion than their soft counterparts. Nonetheless, although soft brushes are typically seen as less abrasive, certain in vitro studies indicate that soft-bristled brushes may inflict greater enamel abrasion than hard-bristled brushes. This is attributed to the greater flexibility of soft bristles, which increases their surface contact with the tooth. Additionally, the denser tuft arrangement of soft brushes tends to hold more toothpaste, which further increases surface contact and, potentially, tooth wear [18]. In response to these concerns, toothbrush design has evolved, incorporating innovations like diverse bristle types and head sizes to minimize enamel abrasion while improving cleaning efficacy.

One recent innovation is the nano-bristle toothbrush, which claims to offer a gentler and more effective cleaning experience by potentially reducing the risk of enamel abrasion. While numerous studies have evaluated the effectiveness of manual toothbrushes in plaque removal, research on the surface roughness caused by different bristle types is limited. Surface roughness is a critical factor in plaque reformation, making it essential to understand how different toothbrush bristles can impact this aspect of oral health. Therefore, the present study was intended to assess the surface abrasion caused by ultra-soft, soft, and recently introduced and unique nano toothbrush bristle designs on the enamel surface at 10 μm × 10 μm at the nanoscale using an atomic force microscope.

AFM is highly recommended for assessing enamel topography due to its ability to provide high-resolution, two-dimensional, and three-dimensional images with minimal sample preparation [19-21]. This makes it an ideal tool for comparing pre- and post-brushing surface roughness on the same sample after exposure to different bristle stiffness levels. AFM's ability to measure surface roughness without altering the sample enhances its suitability for such studies.

This is, to our knowledge, the first AFM investigation comparing surface abrasion induced by various bristle types; thus, the results were juxtaposed with studies employing alternative techniques, such as profilometry, for assessing tooth surface roughness. The findings were analogous to those of the study conducted by Turssi et al., who claimed that the hard-bristle toothbrush transported a greater quantity of abrasive particles beneath the bristle tip, leading to increased abrasive wear in comparison to the soft-bristle toothbrush [22]. Conversely, Alshehab et al. and Wiegand et al. showed that a soft-bristle toothbrush resulted in greater abrasive dentine wear compared to a harsher toothbrush [18,23]. Bizhang et al. posited that the increased flexion of the soft-bristle toothbrush, in contrast to medium or hard-bristle toothbrushes, led to a larger contact area and a more uniform distribution of toothpaste on the dentin surface, thereby resulting in greater abrasive wear [24]. Kumar et al. previously investigated the impact of toothpaste and toothbrushes on enamel abrasion, concluding that soft-bristled toothbrushes, when used with dentifrice, resulted in greater abrasion than hard-bristled toothbrushes [15]. While nano-bristle toothbrushes offer potential benefits in reducing tooth wear, their clinical implications for non-carious cervical lesions, hypersensitivity, heavy-handed brushing, and hand- versus electronic-brush usage require further investigation. In clinical practice, nano-bristle toothbrushes may offer a significant advantage in minimizing enamel erosion and alleviating tooth sensitivity, especially for individuals with inherently softer enamel or those prone to excessive brushing force.

This study, while insightful, had certain limitations. The small sample size and reliance on a single technique to measure surface roughness limit the generalizability of the findings. Future research could benefit from larger sample sizes and the use of additional methods, such as confocal microscopy and histological analysis, for a more comprehensive assessment of surface roughness. Furthermore, as this was an in vitro study, its clinical relevance may be limited. Factors such as saliva composition, individual brushing habits, and variations in temperature and pH levels could influence the results in real-world scenarios. Therefore, future studies involving human participants are necessary to validate these findings and evaluate the clinical efficacy of the nano-bristle toothbrush in terms of both plaque removal and enamel preservation.

## Conclusions

In conclusion, this study highlights the potential impact of different toothbrush bristle types-soft, ultra-soft, and nano-bristle-on enamel surface roughness. Using AFM, we found that nano-bristle toothbrushes caused significantly less enamel abrasion compared to soft and ultra-soft bristle brushes. Despite these findings, the study’s limitations, such as the small sample size and in vitro setting, suggest that further research, particularly involving human subjects and diverse methodologies, is needed to validate the clinical implications of these results. Future studies should explore how toothbrush bristle stiffness interacts with other variables, such as individual oral hygiene practices, to provide a more comprehensive understanding of toothbrush-related enamel abrasion and its long-term effects on oral health.
